# Streptolysin O Deficiency in Streptococcus pyogenes M1T1 *covR/S* Mutant Strain Attenuates Virulence in *In Vitro* and *In Vivo* Infection Models

**DOI:** 10.1128/mbio.03488-22

**Published:** 2023-02-06

**Authors:** Emma L. Langshaw, Simone Reynolds, Victoria Ozberk, Jessica Dooley, Ainslie Calcutt, Mehfuz Zaman, Mark J. Walker, Michael R. Batzloff, Mark R. Davies, Michael F. Good, Manisha Pandey

**Affiliations:** a Institute for Glycomics, Griffith University, Queensland, Australia; b Australian Infectious Diseases Research Centre, School of Chemistry and Molecular Biosciences, The University of Queensland, Brisbane, Queensland, Australia; c Department of Microbiology and Immunology, University of Melbourne at the Peter Doherty Institute for Infection and Immunity, Melbourne, Victoria, Australia; The University of Kansas Medical Center

**Keywords:** *covR/S*, J8CombiVax, SLO, *Streptococcus pyogenes*, dendritic cells, gene expression, neutrophils, skin infection, whole-genome sequencing

## Abstract

Mutation within the Streptococcus pyogenes (*Streptococcus* group A; Strep A) *covR/S* regulatory system has been associated with a hypervirulent phenotype resulting from the upregulation of several virulence factors, including the pore-forming toxin, streptolysin O (SLO). In this study, we utilized a range of *covR/S* mutants, including M1T1 clonal strains (5448 and a *covS* mutant generated through mouse passage designated 5448AP), to investigate the contribution of SLO to the pathogenesis of *covR/S* mutant Strep A disease. Up-regulation of *slo* in 5448AP resulted in increased SLO-mediated hemolysis, decreased dendritic cell (DC) viability post coculture with Strep A, and increased production of tumor necrosis factor (TNF) and monocyte chemoattractant protein 1 (MCP-1) by DCs. Mouse passage of an isogenic 5448 *slo*-deletion mutant resulted in recovery of several *covR/S* mutants within the 5448Δ*slo* background. Passage also introduced mutations in non-*covR/S* genes, but these were considered to have no impact on virulence. Although *slo*-deficient mutants exhibited the characteristic *covR/S*-controlled virulence factor upregulation, these mutants caused increased DC viability with reduced inflammatory cytokine production by infected DCs. *In vivo*, *slo* expression correlated with decreased DC numbers in infected murine skin and significant bacteremia by 3 days postinfection, with severe pathology at the infection site. Conversely, the absence of *slo* in the infecting strain (*covR/S* mutant or wild-type) resulted in detection of DCs in the skin and attenuated virulence in a murine model of pyoderma. *slo*-sufficient and -deficient *covR/S* mutants were susceptible to immune clearance mediated by a combination vaccine consisting of a conserved M protein peptide and a peptide from the CXC chemokine protease SpyCEP.

## INTRODUCTION

The exclusively human pathogen Streptococcus pyogenes (Streptococcus group A-as above; Strep A) causes a variety of illnesses ranging from mild and self-limiting infections such as ‘strep throat’ to invasive life-threatening diseases, including necrotizing fasciitis, which commonly commences with a skin infection. Additionally, the development of post-streptococcal sequelae such as acute rheumatic fever (ARF) and rheumatic heart disease (RHD) contribute significantly to high rates of morbidity and mortality in developing countries and among Indigenous people of developed nations. The burden of ARF and RHD is particularly high in the Indigenous population of Australia, with prevalence rates of RHD reportedly up to 32 cases per 1,000 people (1, 2). Globally, invasive streptococcal diseases and post-streptococcal sequelae are reported to contribute to more than 500,000 deaths annually, with many regions potentially underestimating Strep A-associated deaths (1, 3).

The Strep A M1T1 clone is associated with an increased prevalence of invasive Strep A disease (4). The hypervirulence of this strain is suggested to be due to the ability of this clone to ‘switch’ to an invasive *covR/S* mutant phenotype (5–8). The *covR/S* operon is a two-component negative transduction system that regulates between 10% and 15% of the streptococcal genome (9, 10), the majority of which is involved in virulence factor expression and regulation. When the *covR/S* system acquires a spontaneous mutation, the resultant phenotype is hypervirulent and highly invasive in the host (5–8, 10, 11). This is clearly evidenced in the case of the invasive disease streptococcal toxic shock syndrome (STSS), where studies utilizing genome sequencing have shown that Strep A isolates possessing a mutation within *covR/S* were detected at a frequency of >50% in STSS clinical isolates, but only in 2% of noninvasive isolates (12, 13).

The hypervirulence of *covR/S* mutants may be due to the de-repression of several virulence factors, each of which plays a critical role in immune evasion mechanisms employed by these strains (6). One of these critical virulence factors is streptolysin O (SLO). SLO, a potent pore-forming exotoxin, is a member of the cholesterol-dependent cytolysin family, which includes more than 28 members spanning several bacterial families (14). SLO primarily functions to induce pore formation in target host cells and inhibit phagocytosis, making it an important contributor to Strep A virulence (12, 15–17). Furthermore, the clinical relevance of SLO is underscored by the observation that SLO is commonly overproduced in STSS clinical isolates, which can lead to mortality rates as high as 30% to 70% depending upon the patient’s geographical location and timely access to adequate health care (13, 18).

Host defenses against Strep A infection are initiated by many immune cell types, including dendritic cells (DCs), which are prevalent in the skin and, together with neutrophils, constitute one of the first lines of defense. Strep A have developed several immune evasion mechanisms, including the ability to induce apoptosis of host cells. SLO significantly contributes to this process (15). Given that *covR/S* mutants have a propensity to initiate invasive infections, it might be hypothesized that upregulation of SLO by *covR/S* mutants plays a significant role in this hypervirulent phenotype.

Currently, there is no licensed Strep A vaccine; however, some experimental vaccines are showing promise and are at various stages of preclinical development (19–23). Developing a greater understanding of the complex interactions between host cells and Strep A can only contribute to hastening vaccine development. Such investigations have recently led to the redesign of an existing vaccine candidate, J8-DT/Alum, to provide greater protection against hypervirulent *covR/S* mutant Strep A (11, 24). Immunization with the J8 peptide, which originates from the conserved region of the M protein, conjugated to diphtheria toxoid (DT) as a carrier protein, raises protective opsonic antibodies *in vivo* and is effective in several Strep A challenge models (19, 25). However, the addition of a SpyCEP epitope (S2) to the J8-DT vaccine (herein called J8CombiVax) provides a synergistic protective response better equipped to afford protection against hypervirulent *covR/S* mutant infections (24). This was shown to work by inducing antibodies to negate the streptococcal CXC chemokine-degrading protease, SpyCEP, thus allowing anti-M protein antibodies to interact with neutrophils to kill the *covR/S* mutant Strep A.

With the goal of further understanding the pathogenesis of Strep A, we investigated the relative contribution of SLO in the context of *covR/S* gene regulation. Several different *covR/S* mutant Strep A isolates lacking the SLO gene (*slo*) were evaluated *in vitro* and *in vivo* using a murine model of pyoderma. Our data show that SLO is an essential factor for *covR/S* mutant-mediated hypervirulence.

## RESULTS

### Infection with Strep A 5448AP alters dendritic cell maturation and viability.

**FIG 1:**
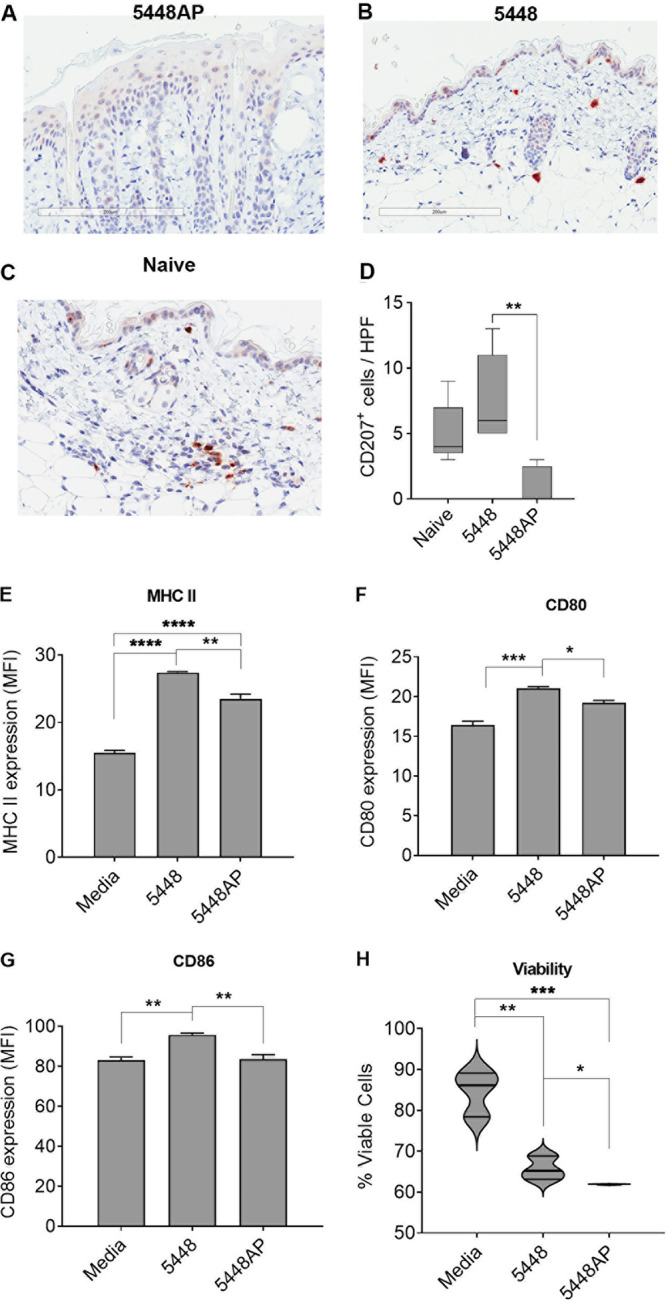
Effect of Streptococcus pyogenes (Strep A) infection on dendritic cell (DC) maturation and viability. Naive BALB/c mice were given a superficial skin infection with (A) 5448AP or (B) 5448 or (C) left uninfected. On day 3 postinfection, skin biopsy sections were stained with antibodies against CD207, scanned, and analyzed using ImageJ software. Representative images are shown; scale bars = 200 μm. (D) CD207^+^ cells in skin sections were enumerated in five high-power fields for three mice/group. Data are presented as box-and-whiskers plot with min/max and median. (E to H) DC2.4 were incubated with 5448 or 5448AP for 12 h *in vitro* and the subsequent expression of maturation marker (E) major histocompatibility complex (MHC) II and co-stimulatory markers (F) CD80 and (G) CD86 was measured by flow cytometry. Data show mean fluorescence intensity (MFI) ± standard error of the mean (SEM) for three biological replicates. (H) A live/dead stain (Thermo Fisher Scientific) was used to assess the viability of cells postinfection in three biological replicates. Data are shown as a violin plot depicting distribution of numerical data. One-way analysis of variance (ANOVA) was used for analysis with Bonferroni’s multiple-comparison test. *, *P* < 0.05; **, *P* < 0.01; ***, *P* < 0.005; ****, *P* < 0.001.

DCs are important mediators of innate and adaptive immunity. Their importance in the context of Strep A infections *in vitro* (16) and following *in vivo* infection (26, 27) has been previously demonstrated. Studies were undertaken to assess their relative contribution in a pyoderma model, where skin abrasion is followed by Strep A inoculation onto the lesion. Naive mice were infected with wild-type M1T1 5448 or *covR/S* mutant M1T1 5448AP, which are known to display differing virulence profiles (7, 8, 28). Skin sections were analyzed via immunohistochemistry (IHC) on day 3 postinfection. Staining the skin sections for CD207 highlighted a lack of skin-specific DCs (Langerhans cells) post-infection with 5448AP (Fig. 1A) compared to postinfection with 5448 (Fig. 1B) or naive skin (Fig. 1C). Quantification of CD207^+^ DCs (via analysis of five separate high-powered fields) on day 3 postinfection demonstrated significantly fewer (*P* < 0.01) DCs in 5448AP-infected skin compared to 5448-infected skin (86% reduction) (Fig. 1D). This observation suggested that the maturation and/or viability of DCs was affected during 5448AP infection. To confirm this observation, DCs from a murine dendritic cell line, DC2.4, were infected with 5448 or 5448AP for 12 h *in vitro*. The subsequent expression of maturation markers (MHC II and co-stimulatory markers CD80 and CD86) was measured via flow cytometry. DC viability postinfection was also assessed using a live/dead stain. DC infection with 5448AP resulted in significantly reduced expression of MHC II, CD80, and CD86 in comparison to infection with 5448 (*P* < 0.05 to 0.01) (Fig. 1E to G, respectively). Infection with either 5448AP or 5448 strains resulted in significantly higher DC death compared to the medium-only control; however, the viability of 5448AP-infected DCs was significantly less than that of 5448-infected DCs. (Fig. 1H). These data show that infection with 5448AP not only results in significantly fewer DCs in the skin but also induces a compromised maturation response in the remaining DCs.

### SLO expression by *covR/S* mutant Strep A strains augments virulence.

**FIG 2:**
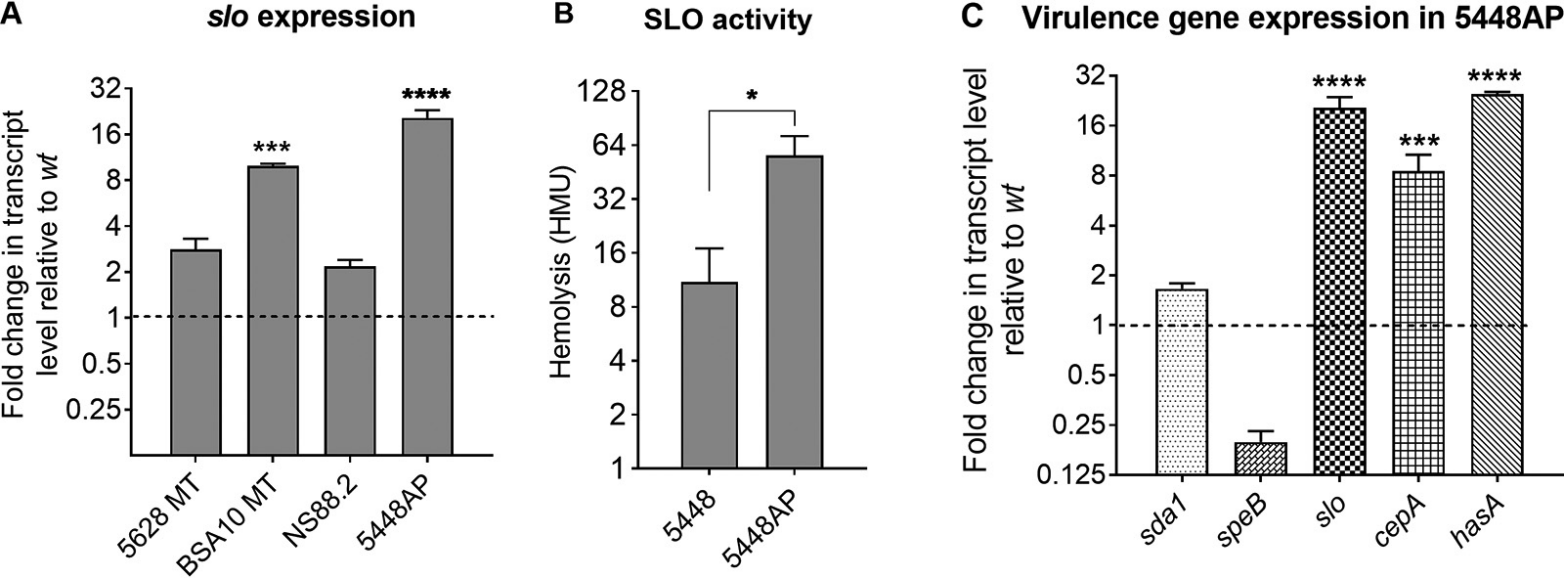
The relative expression and functionality of *slo* by *covR/S* mutant 5448AP. (A) Reverse transcription-PCR (RT-PCR) was performed on mid-log-phase Strep A cultures. Wild-type (WT) values were normalized to a value of 1 for each isogenic pair. Mean transcript values for *slo* mRNA for three biological replicates ± SEM are shown. (B) The mean hemolysis in 0.2 μm-filtered supernatants was assessed via hemoglobin release after 30 min incubation with Strep A. Hemolysis (HMU) was defined as the reciprocal of the supernatant dilution for which hemolysis was greater than 50% compared to the positive Triton-X control. Data from three biological replicates ± SEM are shown. (C) Gene expression of *covR/S*-mediated genes in 5448AP. RT-PCR was performed on mid-log-phase Strep A cultures. Values for 5448 were normalized to a value of 1 for each gene. Mean fold changes in transcript values for three biological replicates ± SEM are shown. One-way ANOVA was used for statistical analyses for each isolate pair with Dunnett’s multiple-comparison test. *, *P* < 0.05; ***, *P* < 0.005; ****, *P* < 0.001.

To investigate the mechanism by which 5448AP might be causing detrimental effects on DC maturation and survival, we first investigated the virulence profile of *covR/S* mutant isolates. A panel of four Strep A isolates and isogenic *covR/S* mutants (representing three *emm* types) was investigated in parallel (Fig. S1A). Reverse transcription-PCR (RT-PCR) was used to assess the relative expression of specific genes (*sda1*, *speB*, *slo*, *cepA*, *hasA*) during mid-log-phase growth. The wild-type (WT) expression of each gene for every isolate was standardized to a value of 1 relative to the housekeeping gene *gyrase A*, which was used as an internal control. All *covR/S* mutants demonstrated upregulated mRNA expression of *slo*, albeit at varying degrees, reflecting the inherent phenotypic differences between strains (Fig. 2A). Expression of *slo* was most strongly upregulated in 5448AP, similar to previous reports in which *slo* is upregulated in a *covR/S* mutant background (4, 6). We also assessed the functional changes in a SLO-mediated red blood cell (RBC) lysis assay using the 5448 and 5448AP strains. Culture supernatants from 5448AP were found to have significantly greater SLO-mediated hemolytic activity compared to supernatants from 5448 (Fig. 2B), which significantly correlated (*P* < 0.01) with the level of *slo* gene expression (Fig. S1B).

10.1128/mbio.03488-22.1 FIG S1 (A) Relative gene expression profiles of *covR/S* mutant Streptococcus pyogenes Strep A isolates. The gene expression profiles of four *covR/S* mutant Strep A isolates and their corresponding *covR/S* wild-types (WT) were assessed using reverse transcription-PCR (RT-PCR) on mid-log-phase cultures. The WT values for each isolate and gene were normalized to a value of 1. Mean transcript values for three biological replicates ± SEM are shown. (B) Correlation between *slo* expression and streptolysin O (SLO) hemolytic activity in 5448AP. The expression of *slo* and the hemolytic activity of 5448AP were measured using RT-PCR and hemolysis assays, respectively. Linear regression and Pearson’s correlation coefficient were calculated using GraphPad Prism software. (C) Hemolytic activity of 5448 mutants. The mean SLO-mediated hemolysis in 0.2 μm-filtered supernatants was assessed via hemoglobin release after a 30-min incubation with Strep A. Hemolysis (HMU) was defined as the reciprocal of the supernatant dilution for which hemolysis was greater than 50% compared to the positive Triton-X control. Data from three biological replicates ± standard error of the mean (SEM) are shown. Two-way analysis of variance (ANOVA) with Dunnett’s multiple-comparison test was used for statistical analysis of gene expression, and a one-way ANOVA with Tukey’s multiple-comparisons test was used for analysis of SLO activity. *, *P* < 0.05; **, *P* < 0.01; ***, *P* < 0.05; ****, *P* < 0.001. Download FIG S1, TIF file, 0.7 MB. Copyright © 2023 Langshaw et al. 2023 Langshaw et al. https://creativecommons.org/licenses/by/4.0/ This content is distributed under the terms of the Creative Commons Attribution 4.0 International license.

We next investigated whether other virulence factors were upregulated in 5448AP. RT-PCR was used to assess the expression of five important virulence genes under the control of *covR/S* in 5448 and 5448AP. We found that the RNA transcripts for SLO (*slo*), SpyCEP (*cepA*), and the hyaluronic acid capsule (*hasA*) were significantly upregulated in 5448AP compared to 5448 (*P* < 0.005 to *P* < 0.001) (Fig. 2C), similar to previous studies (6, 8, 28, 29). This suggested that the enhanced virulence of 5448AP could be a cumulative effect of several virulence factors working in tandem. To further assess SLO-mediated effects in isolation, the chromosomal *slo* knockout strain 5448Δ*slo* strain was employed.

### *slo*-deficient 5448 *covR/S* mutants demonstrate attenuated virulence.

To investigate the role of SLO in the context of *covR/S* mutations, 5448Δ*slo* was passaged through BALB/c mice to select for the *covR/S* mutant phenotype. After the fifth passage, the strain was considered mouse-adapted. From passage five, four individual colonies recovered from spleens were chosen for further characterization. Following sequencing of the *covR/S* operon, all four colonies were identified as *covR/S* mutants with mutations occurring at different locations within the operon (Table S1). 5448Δ*slo covR1* possessed full-length *covR* and *covS* genes with only a substitution mutation at nucleotide (nt) 341 of *covR*. In contrast, the three remaining mutants all possessed mutations within the *covS* gene, each resulting in a premature STOP codon. 5448Δ*slo covS* mutants S1, S2, and S3 (designated 5448Δ*slo covS1*, *covS2*, and *covS3*) possessed a truncated *covS* gene at nt 473, 199, and 9, respectively.

10.1128/mbio.03488-22.5 TABLE S1 5448Δ*slo covR/S* mutants. Download Table S1, PDF file, 0.2 MB. Copyright © 2023 Langshaw et al. 2023 Langshaw et al. https://creativecommons.org/licenses/by/4.0/ This content is distributed under the terms of the Creative Commons Attribution 4.0 International license.

**FIG 3:**
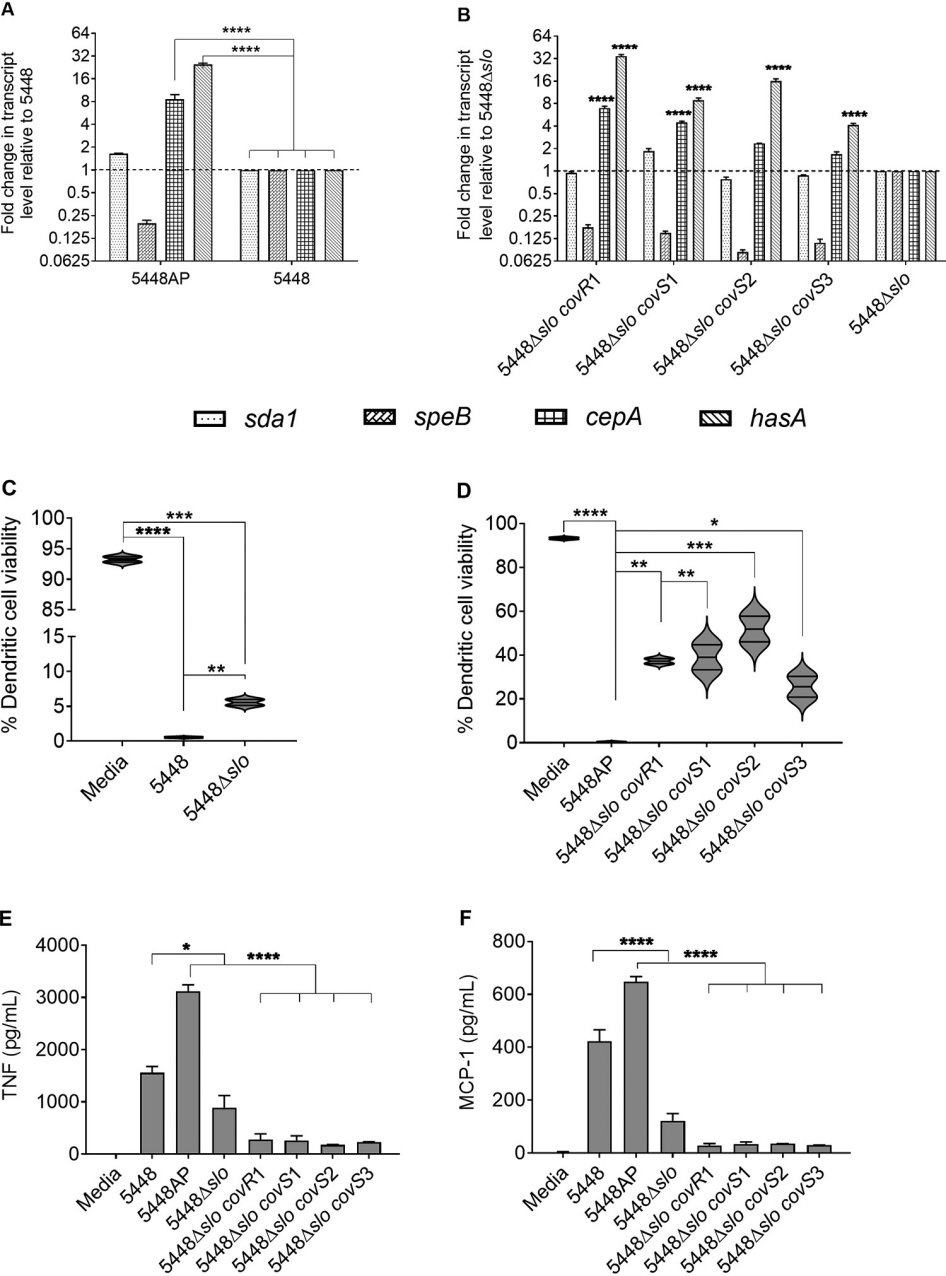
Characterization and interaction of *slo*-deficient 5448 *covR/S* mutant Strep A *in vitro*. (A and B) The gene expression profiles of (A) *slo*-sufficient 5448 and (B) *slo*-deficient 5448 derivatives were assessed using RT-PCR on mid-log-phase Strep A cultures. 5448 values were normalized to 1 for the *slo*-sufficient 5448AP, and 5448Δ*slo* values were normalized to 1 for the *slo*-deficient mutants. Mean transcript values for three biological replicates ± SEM are shown. (C and D) DC2.4 viability postinfection with (C) *covR/S* wild-type 5448 or (D) *covR/S* mutant 5448. DC2.4 was infected with variants of 5448 in the presence and absence of *slo* and *covR/S* mutations. Cellular viability was subsequently assessed via flow cytometry using a live/dead stain (Invitrogen). Data are representative of two separate experiments with three biological replicates each. Mean DC viability ± SEM are shown. (E and F) Tumor necrosis factor (TNF) (E) and monocyte chemoattractant protein 1 (MCP-1) (F) production by 5448Δ*slo covR/S* mutants. Supernatants from the *in vitro* DC2.4 infections were assessed for the presence of pro-inflammatory cytokines using a cytokine bead array. Data are representative of three biological replicates ± SEM. One-way ANOVAs were used for analysis of gene expression and cytokine production, and two-way ANOVAs were used to assess DC viability. *, *P* < 0.05; **, *P* < 0.01; ***, *P* < 0.005; and ****, *P* < 0.001.

Each of the 5448Δ*slo covR/S* mutants (*covR1* and *covS1*-*S3*), as well as the *covR/S* WT parent 5448, 5448AP and 5448Δ*slo*, were assessed for expression of *covR/S*-related virulence factors as described previously. The 5448AP *covR/S* mutant had significantly greater expression of *cepA* and *hasA* transcripts compared to 5448 (*P* < 0.001) (Fig. 3A). Passaging of 5448Δ*slo* induced *covR/S*-mediated gene expression consistent with that of 5448AP (with upregulated *cepA* and *hasA*, and downregulated *speB*) (Fig. 3B). The 5448Δ*slo covS1* strain was most similar to 5448AP in terms of its gene expression profile and *covS* mutation location. Subsequently, 5448Δ*slo covS1* was chosen to undertake *in vivo* studies in mice. The gene expression profile of 5448Δ*slo* was not significantly different from that of 5448 except for *slo* expression. 5448Δ*slo* (and all derivatives generated) possessed no SLO-mediated hemolytic activity *in vitro* (Fig. S1C).

To examine broader genomic changes that may have occurred during animal passage, we performed genome sequencing on 5448, 5448AP, 5448Δ*slo*, and 5448Δ*slo covS1*. Comparative genomics of the four sequenced strains revealed maintenance of overall gene content with the 5448 reference strain (Fig. S2A). One exception was confirmation of the replacement of the *slo* gene with the *cat* resistance gene in the 5448Δ*slo* genomic backgrounds (Fig. S2B). Single-nucleotide polymorphisms (SNP) and indel analysis revealed several SNPs across the genome of the four sequenced strains relative to the 5448 reference genome (CP008776). Two terminating insertions were identified in *covS* following animal passage: a single nucleotide insertion in the animal-passaged 5448AP strain and a two base-pair insertion in the 5448Δ*slo covS1* background. An additional non-synonymous SNP leading to the premature STOP codon was present in the type I restriction endonuclease gene *hsdM* (SP5448_08275) within the 5448Δ*slo* genetic backgrounds (Table S2). *hsd* mutations have previously been shown to alter Strep A transformability, yet do not alter broad transcriptional changes (30, 31).

10.1128/mbio.03488-22.2 FIG S2 Genome sequencing of 5448 strains following animal passage. (A) Genome ring of 5448 reference genome (CP008776) showing GC plot; GC skew; the relative location of 5448 prophage, ribosomal and repeat sequences (red blocks). The 4 outside rings refer to BlastN homology of Illumina genome assemblies of 5448, 5448AP, 5448Δ*slo*, and 5448Δ*slo covS1* relative to the 5448 reference genome sequence. Location of streptolysin O (*slo*) gene is annotated. Figure was generated using BRIG (45) (B) Comparative BlastN alignment of the streptolysin O genomic regions from 5448 reference genome (CP008776) and the 4 mutant strains analyzed in this study, confirming that *slo* was successfully replaced with the chloramphenicol acetyltransferase resistance marker (*cat*). Nucleotide sequence identity is graded from blue to red. Image was generated using EasyFig (46). Download FIG S2, JPG file, 2.3 MB. Copyright © 2023 Langshaw et al. 2023 Langshaw et al. https://creativecommons.org/licenses/by/4.0/ This content is distributed under the terms of the Creative Commons Attribution 4.0 International license.

10.1128/mbio.03488-22.6 TABLE S2 SNP mutations in 5448. Download Table S2, PDF file, 0.2 MB. Copyright © 2023 Langshaw et al. 2023 Langshaw et al. https://creativecommons.org/licenses/by/4.0/ This content is distributed under the terms of the Creative Commons Attribution 4.0 International license.

The virulence of *slo*-deficient 5448 and *covR/S* mutant strains was further assessed utilizing the DC2.4 murine cell line. DCs were infected *in vitro* with each of the 5448 variants for 12 h, and cell viability was assessed with flow cytometry using a live/dead stain. A comparison of DC viability between isogenic 5448 and 5448Δ*slo* demonstrated significantly higher numbers of live DCs when they were infected with 5448Δ*slo* (*P* < 0.05) (Fig. 3C). Similarly, DCs infected with the passaged *slo* knockout *covR/S* mutants were significantly more viable (*P* < 0.01 to *P* < 0.001) than DCs infected with *slo*-sufficient *covR/S* mutants (Fig. 3D). In all cases, the presence of *slo* was shown to play a critical role in the virulence mechanism of *covR/S* mutant strains.

To assess whether there was a difference in pro-inflammatory cytokine abundance in the supernatants of infected DCs, we utilized a cytokine bead array. The cytokine production was normalized to the same number of viable DCs across all cohorts. We found significantly greater amounts of tumor necrosis factor (TNF) and monocyte chemoattractant protein 1 (MCP-1) (Fig. 3E and F, respectively) produced by DCs infected with 5448 and 5448AP (both expressing *slo*) in comparison to their *slo*-deficient derivatives (*P* < 0.05). Significantly higher production of TNF and MCP-1 (*P* < 0.05 to 0.001) by 5448AP-infected DCs was consistent with the SLO expression levels and activity seen with 5448AP (Fig. 2A and B). Furthermore, these data demonstrated a role of SLO, in a *covR/S* mutant phenotype, in the modulation of pro-inflammatory cytokine responses.

Next, to assess the *in vivo* effect of SLO on the skin-resident DCs in mice, we performed IHC analyses. Naive BALB/c mice were infected with 5448, 5448AP, 5448Δ*slo*, or 5448Δ*slo covS1* via the superficial skin infection method and skin samples were assessed on day 3. Although DC presence was evident in both 5448Δ*slo* and 5448Δ*slo covS1*-infected cohorts (Fig. S3A and B), the numbers of DCs in both *slo*-deficient cohorts were significantly greater compared to those in 5448AP-infected skin (*P* < 0.005). These observations were consistent with *in vitro* data (Fig. 3C and D) in which SLO-deficient mutants were shown to be significantly less detrimental to the survival of DCs. These data highlighted that SLO plays an important role in DC viability at the skin infection site. There was no significant difference in the number of DCs in 5448Δ*slo*-infected or 5448Δ*slo covS1*-infected skin (Fig. S3C), further implying SLO-mediated virulence mechanisms in skin infection.

10.1128/mbio.03488-22.3 FIG S3 CD207^+^ dendritic cells in infected murine skin. Naive BALB/c mice were infected with (A) 5448Δ*slo* or (B) 5448Δ*slo covS1* and skin sections were analyzed via immunohistochemistry on day 3. Scale bars = 200 μm. (C) CD207^+^ cells were enumerated in five high-powered fields for three mice/group and compared with 5448AP. Data are presented as box-and-whiskers plots: line in the box indicates the median, box extremities indicate upper and lower quartiles, and whiskers show minimum to the maximum values. A one-way ANOVA with Tukey’s multiple-comparison test was used to assess statistical significance. **, *P* < 0.01; ***, *P* < 0.005. Download FIG S3, TIF file, 1.8 MB. Copyright © 2023 Langshaw et al. 2023 Langshaw et al. https://creativecommons.org/licenses/by/4.0/ This content is distributed under the terms of the Creative Commons Attribution 4.0 International license.

**FIG 4:**
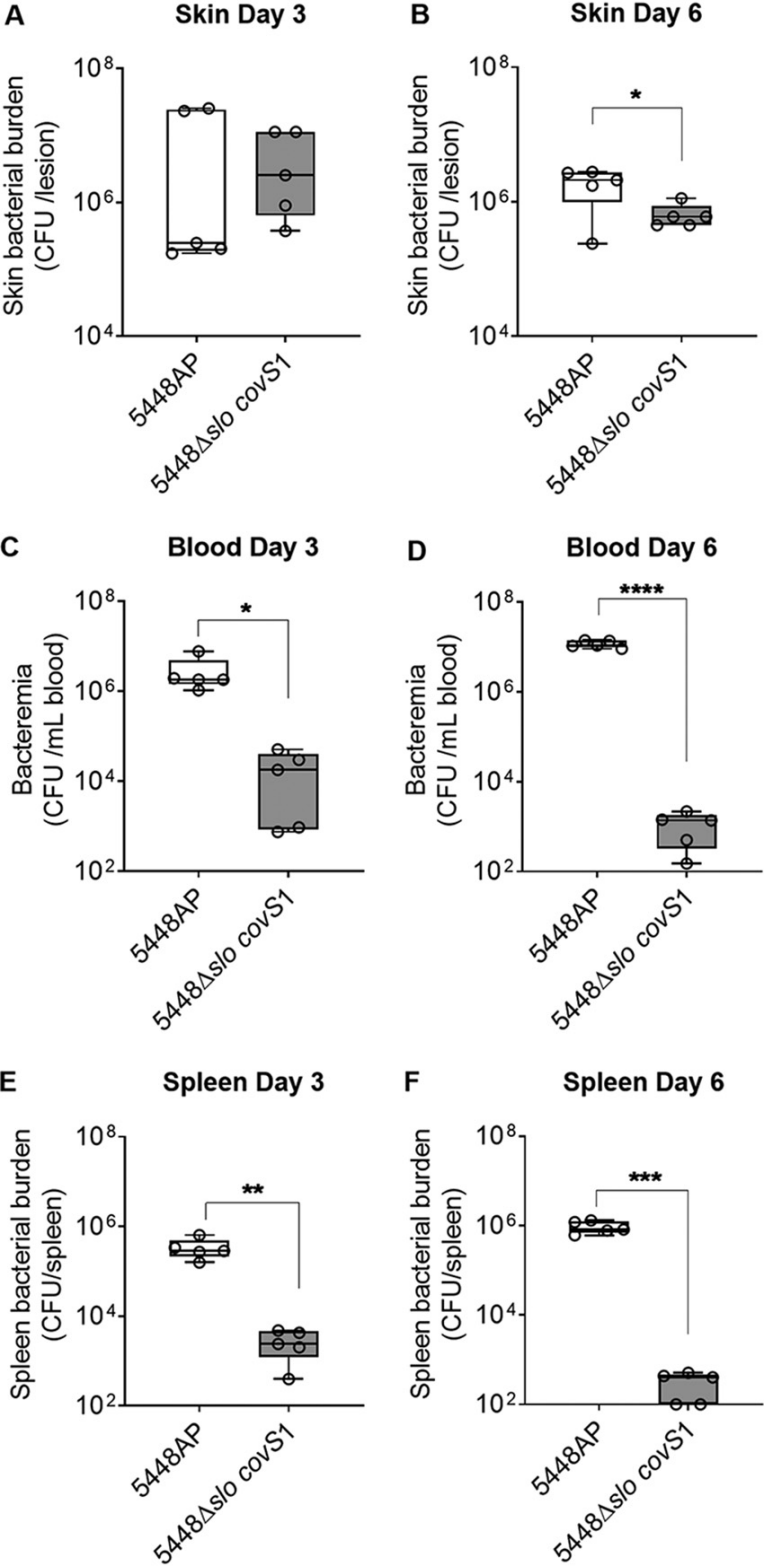
Virulence of 5448 *covR/S* mutant with or without *slo* in a skin challenge model. Naive BALB/c mice (female, 4 to 6 weeks old) were infected with Strep A 5448AP and 5448*Δslo covS1* via the superficial skin infection method. (A and B) skin, (C and D) blood, and (E and F) spleen samples were collected at designated time points and processed to assess local (skin) and systemic (spleen and blood) StrepA burdens. Figures show the Strep A burden for 5 mice/group/time point as box-and-whiskers plots, minimum to maximum, with each dot representing one mouse. Student’s *t* tests were used for statistical analyses. *, *P* < 0.05; **, *P* < 0.01; ***, *P* < 0.005; and ****, *P* < 0.001.

To assess the *in vivo* virulence capacity of the *slo*-sufficient and -deficient *covR/S* mutants, naive BALB/c mice were infected with 5448AP or 5448Δ*slo covS1* using the superficial skin infection method (11). The skin biopsy specimens taken at day 3 postinfection showed that mice infected with the *slo*-sufficient strain 5448AP had more severe pathology at the infection site compared to mice with *slo*-deficient infection (Fig. S4A and B, respectively). Both cohorts demonstrated comparable skin Strep A burdens on day 3 (Fig. 4A). However, by day 6, the Strep A burden in the skin of mice infected with the 5448Δ*slo covS1* strain was significantly less than that seen in mice infected with the *slo*-sufficient 5448AP (*P* < 0.05) (Fig. 4B). Furthermore, a significantly enhanced invasive ability of the *slo*-sufficient *covR/S* mutant 5448AP was evident in the blood (Fig. 4C and D) and spleen (Fig. 4E and F) samples taken on days 3 and 6 postinfection.

10.1128/mbio.03488-22.4 FIG S4 Skin lesions on infected mice. (A and B) Naive BALB/c mice (female, 4 to 6 weeks, *n* = 10/group) were infected with (A) 5448AP or (B) 5448Δ*slo covS1* via the skin route. Images show infected wounds at day 3 postinfection and are representative of each infected cohort. Lesion size at the infection site was also assessed for J8CombiVax-immunized and non-immunized mice infected with 5448AP or 5448Δ*slo covS1* via the skin. (C and D) At days 3 (C) and 6 (D) postinfection, infected lesion size was measured for each mouse and then lesions were excised for Strep A quantification. A one-way ANOVA with Tukey’s multiple-comparison test was used for statistical analysis. *, *P* < 0.05; ***, *P* < 0.005. Download FIG S4, TIF file, 1.1 MB. Copyright © 2023 Langshaw et al. 2023 Langshaw et al. https://creativecommons.org/licenses/by/4.0/ This content is distributed under the terms of the Creative Commons Attribution 4.0 International license.

### Evasion of neutrophil killing is independent of SLO.

The ability of each 5448 isolate to evade killing by human neutrophils was assessed. Neutrophils were isolated from three healthy volunteers and incubated with Strep A at an MOI of 1:10 (Strep A:neutrophils). Following an incubation period of 1 h, bacterial survival in the presence of human neutrophils was assessed.

**FIG 5:**
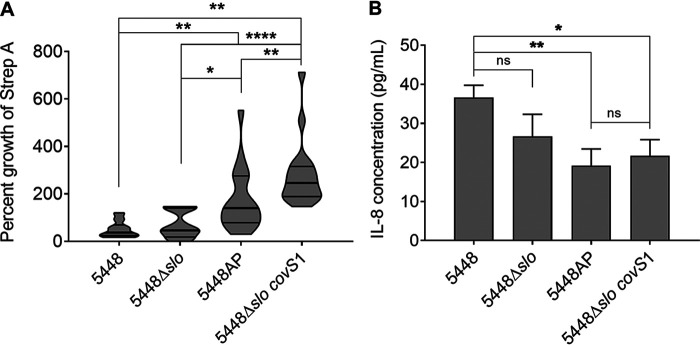
Streptolysin O (SLO) interaction with human neutrophils. (A) Killing of Strep A by human neutrophils. Neutrophils were isolated from the blood of three healthy volunteers using Polymorph Prep (Axis-Shield). The neutrophils were infected with Strep A at a ratio of 1:10 (Strep A: neutrophils) in RPMI for 1 h at 37°C ± 5% CO_2_. Strep A colonies were enumerated by plating on blood agar. Data show Strep A percent growth for three individual assays from three donors as a violin plot depicting distribution of numerical data. (B) Measurement of residual interleukin (IL)-8 in the supernatants of Strep A and human neutrophil coculture. A cytokine bead array was used to quantify IL-8 in the supernatants from *in vitro* neutrophil killing assay infections. Data show mean cytokine concentration for three donors ± SEM. One-way ANOVA with Tukey’s multiple-comparison test were used for statistical analysis. *, *P* < 0.05; **, *P* < 0.01; ****, *P* < 0.001.

The data demonstrated that at the MOI of 0.1, each of the three donors’ neutrophils killed 5448 and 5448Δ*slo* to a similar degree (survival ranged from 50% to 70%) (Fig. 5A). Both *covR/S* mutant organisms (5448AP and 5448Δ*slo covS1*) grew significantly better in the presence of neutrophils compared to their corresponding WT isolates (*P* < 0.01 and 0.001, respectively). Notably, a lack of SLO on a *covR/S* phenotype (5448Δ*slo covS1*) led to significantly higher evasion of neutrophil killing compared to that of a SLO-sufficient isolate (5448AP).

We observed an inverse correlation between Strep A survival and residual interleukin (IL)-8 produced by the neutrophils. A significantly greater amount of IL-8 was detected when the neutrophils were infected with 5448 compared to when they were infected with 5448AP (*P* < 0.01) (Fig. 5B), consistent with the upregulation of SpyCEP mRNA by 5448AP. This was also significantly higher than that in 5448Δ*slo covS1* (*P* < 0.05). IL-8 degradation caused by both the *slo*-deficient and -sufficient 5448 *covR/S* mutants was comparable (Fig. 5B). This result, and data showing that IL-8 degradation was similar in *slo*-sufficient and -deficient WT Strep A, suggests that the enhanced growth of SLO-deficient *covR/S* Strep A in the presence of human neutrophils is not linked to the strain’s ability to degrade IL-8.

### Vaccination with J8CombiVax compensates for neutrophil paucity and protects against *covR/S* mutant skin infection.

Neutrophils and DCs are both critical for the adequate control of Strep A skin infection (11, 12, 16). Our *in vitro* and *in vivo* data suggested that SLO is responsible for an altered virulence of Strep A strains. Therefore, to assess the role of SLO in vaccine-mediated immunity, we performed skin challenges in which the protective efficacy of J8CombiVax (J8-DT+K4S2-DT/Alum) was assessed against *slo*-sufficient and -deficient 5448 *covR/S* mutants using the murine model of skin infection.

**FIG 6:**
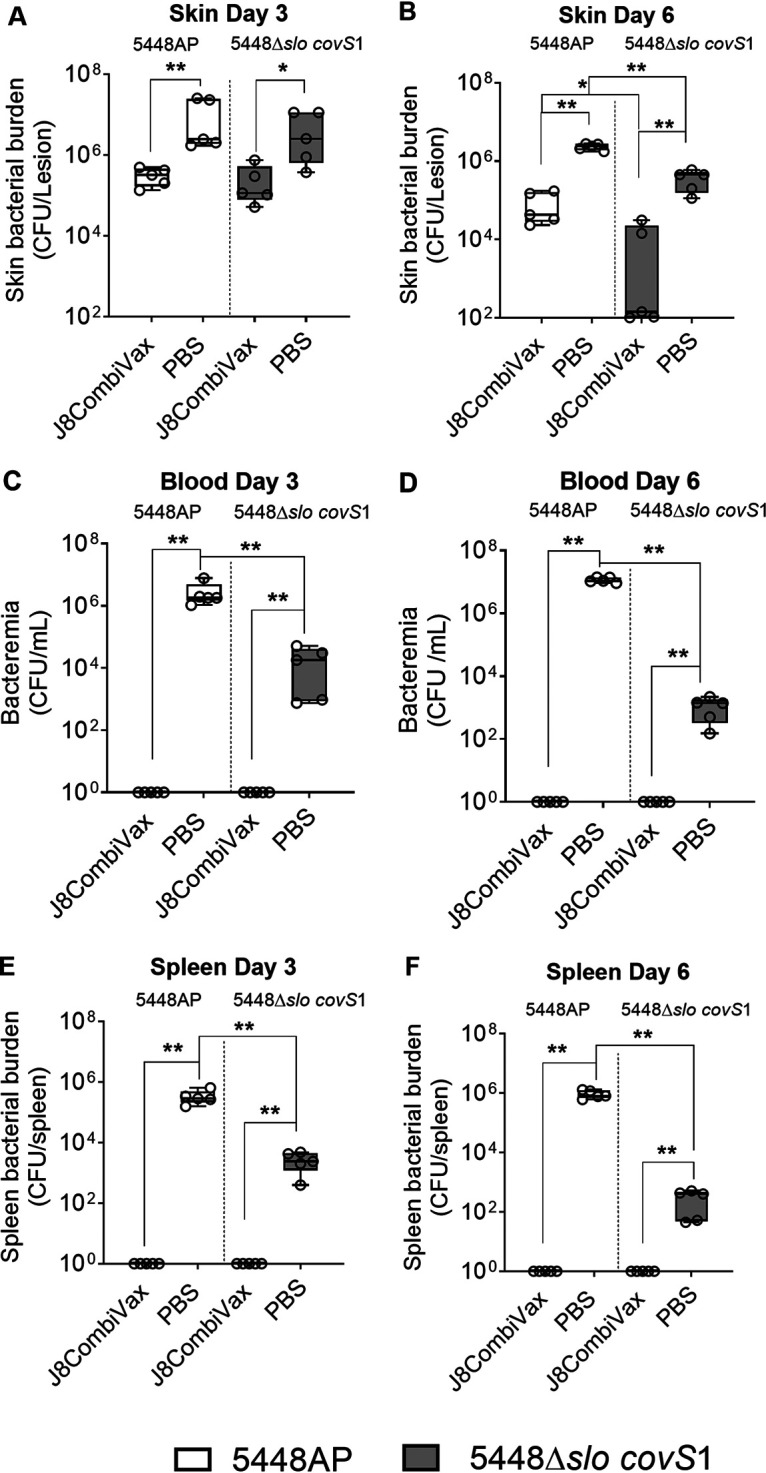
Protective efficacy of J8CombiVax against Strep A infection. BALB/c mice (female, 4 to 6 weeks old; *n* = 10/group) were immunized with the J8CombiVax (J8-DT + K4S2-DT/Alum, 30 μg of each antigen) or phosphate-buffered saline on days 0, 21, and 28. The immunized and non-immunized mice were infected with Strep A 5448AP and 5448*Δslo covS1* via the superficial skin infection method. At designated time points (days 3 and 6) skin, blood, and spleen samples were collected and processed to assess local (skin) and systemic (spleen and blood) Strep A burdens. Figures show the bacterial burden in (A and B) skin, (C and D) blood, and (E and F) spleen for 5 mice at each designated time point. Data are presented as box-and-whiskers plots, minimum to maximum, with each dot representing one mouse. Student’s *t* tests were used for statistical analyses. *, *P* < 0.05; **, *P* < 0.01.

BALB/c mice were immunized with J8CombiVax, then infected with 2 × 10^6^ CFU of 5448AP or 5448Δ*slo covS1* via the superficial skin infection method. On day 3 postinfection the vaccinated mice infected with *slo*-sufficient 5448AP or *slo*-deficient 5448Δ*slo covS1* both had significantly reduced skin Strep A burdens compared to their corresponding non-vaccinated controls (Fig. 6A). The mice infected with the 5448Δ*slo covS1* strain had significantly smaller lesions at the infection site compared to mice infected with the *slo*-sufficient *covR/S* mutant 5448AP, irrespective of immunization status (*P* < 0.05 to 0.005) (Fig. S4C). By day 6 postinfection, vaccinated mice challenged with 5448Δ*slo covS1* demonstrated a significantly greater reduction in skin Strep A burden compared to vaccinated mice challenged with the *slo*-sufficient 5448AP (*P* < 0.05) (Fig. 6B). There was no significant difference in lesion size at day 6 postinfection between all infected cohorts, although there was a trend of smaller lesions in mice challenged with *slo*-deficient Strep A (Fig. S4D). Strep A was not found in the blood or spleen of vaccinated mice following challenge with either 5448AP or 5448Δ*slo covS1* (Fig. 6C to F). The vaccine provided complete systemic protection against both isolates. However, in non-vaccinated mice, a significantly lower Strep A burden (*P* < 0.01) was noted for 5448Δ*slo covS1* in comparison to 5448AP, further demonstrating the attenuated invasive capability of *slo*-deficient 5448AP (Fig. 6C to F). Taken together, these data demonstrate that the altered virulence of *slo*-deficient 5448Δ*slo covS1* neither compromised nor augmented the efficacy of J8CombiVax against systemic infection.

## DISCUSSION

Strep A *covR/S* mutants have an increased invasive propensity due to their ability to circumvent the host immune response and invade deep tissue sites (7, 32). The hypervirulence of these strains contributes to severe Strep A infections worldwide. M1T1 strains isolated from invasive infections are often associated with *covR/S* mutation acquisition, and M1T1 is also overrepresented in invasive disease isolates (4, 32, 33). Understanding the complexities of the host-pathogen interaction is vital to the advancement of infection treatment and control and the development of effective vaccines.

DCs are among the first responders at the infection site and thereby represent a crucial part of the innate immune response against streptococcal infections. A previous study highlighted the importance of DCs in immune responses against Strep A infection, with effective DC maturation being essential to this process (26). In this study, we observed that M1T1 5448AP induced less maturation of DCs *in vitro* than its *covR/S* WT counterpart, as indicated by the downregulation of MHC II, CD80, and CD86. CD86 is regarded as a marker of early DC maturation, with implications in initiation of immune responses by T cells; whereas CD80, being expressed on fully mature DCs, may function to amplify the immune response once it is initiated (34, 35). A study by Borriello et al. (34) demonstrated that mice deficient in CD86 expression were greatly limited in their ability to induce a T-helper response and also presented a more severe immunodeficient phenotype. Not only do appropriate T cell responses enable the induction of high-affinity antibodies and antibody-producing B cells, but they also provide survival signals for the maintenance of memory B cells (36–38). Cytokines produced by T cells are responsible for the class-switching recombination event that distinguishes different immunoglobulin classes (38). In conclusion, appropriate and timely DC and T cell responses are essential for effective host immunity against Strep A.

Here, we hypothesized that the upregulated expression of *slo* by the *covR/S* mutant 5448AP was primarily responsible for this altered DC response. The murine histology data supported this notion, with *slo*-deficient 5448Δ*slo covS1* not inducing the same level of DC death at the infection site compared to the *slo*-sufficient 5448AP strain. This validates other studies in which Strep A isolates expressing high levels of SLO induced decreased maturation and apoptosis (as defined by the presence of hypodiploid nuclei) of DCs (16). Cortes and Wessels (16) also found that *slo*-expressing Strep A induced greater caspase production by infected DCs compared to *slo*-deficient strains. In our studies, we saw an upregulation of TNF by DCs. It is possible that 5448AP induces DC apoptosis via SLO-mediated TNF production which consequently leads to caspase activation and, ultimately, to DC death.

In several studies, *slo* expression has been linked to the impairment of phagocytes (12, 15, 17). However, in this study, we show that *slo* expression is important in the context of *covR/S* mutations for cellular interactions, potentially in partnership with the hyaluronic acid (HA) capsule. Several studies have highlighted a relationship between these two virulence factors, with its effects implicated in increased LL-37-induced resistance to killing by human cells (39) and altered cellular maturation responses (16), among others.

The relationship between SLO and the HA capsule can potentially be seen in the neutrophil interaction studies in which the *covR/S* mutants lacking *slo* (with significant upregulation of *hasA* compared to 5448Δ*slo*) survived better in the presence of human neutrophils. This is in comparison to 5448Δ*slo* (no significant difference in *hasA* gene expression compared to 5448), which was effectively killed by human neutrophils. The increased capsule production is likely responsible for the ineffective neutrophil killing in the absence of *slo*. Future studies would benefit from investigating a *covR/S* mutant double-knockout with *slo* and *hasA*. Furthermore, we observed a significantly greater amount of neutrophil-derived IL-8 produced after neutrophils were infected with 5448 compared to that after infection with 5448AP. This might be attributed to the significant increase in SpyCEP gene expression (*cepA*) by 5448 upon acquisition of the *covR/S* mutation, as seen in our gene expression studies and the findings of previous studies (40).

In addition to the altered *in vitro* responses, the 5448Δ*slo covS1* strain also demonstrated a diminished ability to disseminate from a superficial skin wound to cause systemic infections in a murine model. Furthermore, 5448Δ*slo covS1* produced less severe wounds at the infection site and significantly less bacteremia. This suggests that Strep A requires *slo* to invade and subsequently survive within sterile sites of the host, such as the blood. This observation is supported by a study by Zhu et al. (17) that highlighted the inability of *slo* mutants to cause cytotoxicity in keratinocytes. In the present study, we observed that the 5448Δ*slo covS1* strain resisted phagocytosis by human neutrophils *in vitro* but exhibited less virulence in the murine model of skin infection. This could be attributed to the inherent differences in cellular interactions *in vivo* compared to those modeled *in vitro*, where the intricate host immune systems are difficult to replicate. In addition, the differential behavior of mouse-adapted Strep A strains with human and mouse immune systems may also be contributing to this outcome. While these *in vitro* interaction studies are valuable for providing indications of virulence mechanisms, the utilization of murine models generally provides a more accurate depiction of host infections. Although the genomic sequencing confirmed that none of the observed differences between the SLO-deficient *covR/S* wild-type and mutant strain would have impacted the observed phenotype, it is possible that differential expression of other virulence factors may be contributing to overall virulence. Nevertheless, vaccination with J8CombiVax was able to effectively protect mice from infection with *slo* knockout strains despite their altered virulence (exhibiting upregulation of several *covR/S* mutation-mediated virulence factors).

One caveat of our study was the use of non-isogenic Δ*slo covR/S* WT and *covR/S* mutant isolates (5448Δ*slo* and 5448Δ*slo covS1*). *covR/S* mutation in 5448Δ*slo* was achieved through animal passaging, and it was not clear whether additional mutations were accrued during passage or whether they were manifested in the phenotypes observed. Whole-genome sequencing (WGS) addressed these concerns, confirming that the phenotypes observed in this study can be attributed to the *slo* gene and not to mutations in other genes introduced through animal passage. WGS of the strains investigated in this study identified expected mutations in the *covS* gene and an additional mutation of note to the *hsdM* gene. In *covS* (SP5448 08090), two terminating insertions were identified following animal passage: a single nucleotide insertion in the 5448AP strain (position 1,543,451 in 5448) and a two base-pair insertion in 5448Δ*slo covS1* (position 1,543,856 in 5448). As has been demonstrated in previous studies, passaged derivatives of a parent strain can exhibit multiple different *covS* mutations, including amino acid substitution and truncations, and these mutations have been shown to be responsible for virulence modulation (6, 7).

An additional non-synonymous SNP leading to the premature STOP codon was present in the type I restriction endonuclease gene *hsdM* (SP5448_08275) within the 5448Δ*slo* genetic backgrounds. *hsdM* is part of a type 1 restriction-modification system comprised of a three-gene cluster with separate restriction endonuclease (*hsdR*), specificity (*hsdS*), and methyltransferase (*hsdM*). The type 1 restriction-modification system has a role in DNA methylation. Investigations by others on the role of this system in gene expression and virulence determined that the type I restriction-modification system has an effect in protection against exogenous DNA (30), and that mutations (spontaneous or introduced) to *hsdM* within this system can be exploited to increase transformability in a strain, with no obvious change in growth or off-target gene expression (31). The outcomes of these studies suggest that the *hsdM* mutation in 5448Δ*slo* was positively selected for during the transformation process and this mutation was then maintained in the passaged derivative 5448Δ*slo covS1*. A study by Finn et al. (30) also suggests that this mutation has had no impact on off-target gene expression.

Overall, this work demonstrates that upregulation of *covR/S* mutation-mediated virulence factor expression is not sufficient to overcome virulence attenuation due to the absence of *slo*. These studies also highlight the role of DCs and neutrophils in the interaction with Strep A during infection and suggest that *slo* expression influences Strep A pathogenesis. SLO is secreted by nearly all Strep A isolates and the findings from this study will therefore have far-reaching implications. Taken together, these data underscore the hypothesis that *covR/S* mutation-mediated virulence is dictated, in part, by *slo*.

## MATERIALS AND METHODS

### Strep A strains and growth conditions.

M1T1 clone 5448 and 5448AP have been previously described (4). Victor Nizet kindly provided 5448Δ*slo* (15). All 5448Δ*slo covR/S* mutant derivatives were generated via sequential intraperitoneal passaging through BALB/c mice. Strep A strains were grown overnight at 37°C on Columbia blood agar (Oxoid, Australia) supplemented with 5% defibrinated horse blood (Equicell, Australia). Single colonies were used to inoculate Todd Hewitt broth (Oxoid, Australia) supplemented with 1% yeast extract (Oxoid) and 1% neopeptone (Difco, Australia). Bacterial cultures were grown to stationary phase (16 h) or mid-log phase (OD_600_ [optical density at 600 nm] = 0.4) at 37°C for specific assays.

### SLO activity assay.

The SLO activity assay was adapted from previous methods (41, 42). Stationary-phase Strep A cultures (normalized to OD_600_ = 1.0) were centrifuged for 10 min at 845 × *g*. Dithiothreitol was added to 0.2 μm-filtered supernatants to a final concentration of 4 mM and incubated for 10 min at room temperature. Supernatants were serially diluted 1:2 in phosphate-buffered saline (PBS) in a 96-well plate. Sheep red blood cells (SRBC, 2% vol/vol, Innovative Research) were added to all wells and incubated for 30 min at 37°C + 5% CO_2_. The plate was centrifuged for 10 min at 845 × *g*, and hemoglobin release in the supernatant was measured at 450 nm. Triton X-100 (1% vol/vol in PBS, Thermo Fisher Scientific, Australia) and PBS alone were mixed with erythrocytes for the positive and negative controls, respectively. Cholesterol (25 μg/mL; Sigma-Aldrich, Australia) was added to Strep A culture supernatant and erythrocytes as a SLO-specific inhibitor, whereas trypan blue (50 μg/mL; Gibco, Australia) was used as an SLS-specific inhibitor. The number of hemolytic units/mL corresponded to the reciprocal of the dilution of supernatant that yielded 50% lysis of the erythrocyte suspension, where 100% corresponds to that caused by 1% Triton X-100 (Thermo Fisher Scientific, Australia).

### DC2.4 dendritic cell infection assays.

The murine bone marrow-derived dendritic cell line, DC2.4, was cultured in RPMI 1640 medium (pH 7.4) (Gibco) supplemented with 10% fetal calf serum (FCS, Thermo Fisher Scientific, Australia), 1% pen-strep (Gibco), 1% l-glutamine (Gibco), and 0.1% 2-mercaptoethanol (Gibco) at 37°C + 5% CO_2_. Cells were passaged once adherent cells were 90% confluent, approximately every 3 days.

For DC activation and maturation studies, dendritic cells were harvested from culture and incubated with Strep A at an MOI of 2:1 or 10:1 (Strep A:DC). Lipopolysaccharide (2 μg/mL, Sigma-Aldrich, Australia) was used as a positive control, with DCs in medium alone used as the negative control. DC/Strep A cultures were incubated without antibiotics for 12 h at 37°C + 5% CO_2_. Cells were collected via centrifugation and supernatants were stored at −80°C for cytokine analyses. Cells were resuspended in Fc block (CD16/CD32; BD Pharminogen, Australia) and incubated on ice for 10 min to prevent nonspecific binding of antibodies. The LIVE/DEAD Fixable Cell Stain (Life Technologies, Australia) was used according to manufacturer’s instructions. For DC maturation assessment, fluorochrome-conjugated antibodies were added in a 1:100 dilution in PBS + 1% bovine serum albumin and incubated on ice for 40 min protected from light. The antibody cocktail used included anti-mouse/rat major histocompatibility complex (MHC) II (I-Ek) fluorescein isothiocyanate (Affymetrix eBioscience), rat anti-mouse CD86 (GL-1) PE (BD Pharminogen, Australia), and hamster anti-mouse CD80 APC (16-10A1; BD Pharminogen, Australia) with the appropriate isotype controls. Samples were analyzed using a CyAn ADP flow cytometer (Beckman Coulter) employing Summit software v4.3 (Beckman Coulter).

### Cytometric bead array.

Cytometric bead arrays (CBA; Becton, Dickinson and Co.) were performed to quantify secreted cytokines. Each CBA was used as per manufacturer’s instructions. All samples and standards were run on a CyAn ADP flow cytometer (Beckman Coulter). Cytokine quantifications were calculated using FCAP Array software v3.0 (Becton, Dickinson and Co.).

### Gene expression analysis using RT-PCR.

RNA was extracted from mid-log-phase cultures (OD_600_ = 0.4) using the phenol-chloroform extraction method detailed in the TRIzol Plus RNA Purification kit and then purified as per the manufacturer’s instructions. (Invitrogen, Australia) RNA samples were treated with RNase-free DNase I (Roche, Australia) for 20 min at 37°C, and the reaction was stopped with the addition of 0.2 M EDTA (pH 8.0) to a final concentration of 8 mM. cDNA was synthesized from 75 ng RNA using iScript Reverse Transcription Supermix for RT-PCR (Bio-Rad, Hercules, CA, USA). RT-PCR was performed using iQ SYBR Green Supermix (Bio-Rad) incorporating 0.2 μM of each primer (primer sequences provided in Table S3). The thermal profile consisted of 95°C for 3 min; 45 cycles of 95°C for 15 sec, 60°C for 1 min, and 90°C for 10 sec; and a melt curve (55°C to 95°C with 0.5°C-increments/step).

10.1128/mbio.03488-22.7 TABLE S3 RT-PCR primers. Download Table S3, PDF file, 0.1 MB. Copyright © 2023 Langshaw et al. 2023 Langshaw et al. https://creativecommons.org/licenses/by/4.0/ This content is distributed under the terms of the Creative Commons Attribution 4.0 International license.

The relative amounts of gene-specific cDNA were quantified using the threshold cycle (ΔΔ*CT*) method, where *gyrA* amplification was used as the internal control. The fold change in transcript level was compared to that of *gyrA*, and all *covR/S* WT values were normalized to a baseline value of 1. The amplification efficiency was between 90% and 110% for each gene as determined by standard curve analysis over several dilutions.

### *covR/S* gene sequencing.

*covR/S* sequencing was conducted as previously described (7). Briefly, InstaGene Matrix (Bio-Rad, Australia) was used to extract genomic DNA from Strep A colonies as per the manufacturer’s instructions. PCR was used to amplify the entire *covR/S* operon. Each PCR was prepared using a master mix containing 1× GoTaq Mastermix (Promega, Australia), 0.2 μM of each primer (*covRS* 1F and *covRS* 12R, primers listed in Table S4), 50 ng template DNA, and sterile MilliQ H_2_O made up to a final volume of 50 μL. The thermal profile consisted of an initial denaturation step of 95°C for 2 min; 35 cycles of 95°C for 1 min, 60°C for 1 min, and 72°C for 1 min; then a final elongation step of 72°C for 5 min. Each amplicon was purified using the High Pure PCR Clean-Up Micro kit (Roche, Australia) following the manufacturer’s instructions. Purified DNA was sequenced commercially at the Australian Genome Research Facility (Queensland, Australia).

10.1128/mbio.03488-22.8 TABLE S4 Sequencing primers. Download Table S4, PDF file, 0.1 MB. Copyright © 2023 Langshaw et al. 2023 Langshaw et al. https://creativecommons.org/licenses/by/4.0/ This content is distributed under the terms of the Creative Commons Attribution 4.0 International license.

### Whole-genome sequencing.

Genome sequencing of 5448, 5448AP, 5448Δ*slo*, and 5448Δ*slo covS1* Strep A strains was performed on the Illumina NextSeq 500 platform with 150-bp paired-end chemistry (Doherty Applied Microbial Genomics, University of Melbourne Australia). Sequence reads were processed with the Nullarbor v2.0 pipeline (https://github.com/tseemann/nullarbor) and mapped to the 5448 complete reference genome (GenBank ID: CP008776). SNPs and small indels were identified using 17/1/23 17:22 ArtID: 03488-22 DOI:10.1128/mbio.03488-22 CE: rar Contribution of SLO to covR/S Mutant Strep A Virulence mBio Month YYYY Volume XX Issue XX 10.1128/mbio.03488-22 13 BWA-MEM v0.7.17 as part of snippy v4.6.0 (github/tseemann/snippy, v4.0).

### Neutrophil killing assays.

Neutrophils were isolated from human blood using Polymorph Prep (Axis Shield, ELITech, Australia). Approval was granted by the Griffith University Human Research Ethics Committee (GU HREC no. BDD/01/15/HREC). Written informed consent was obtained from all donors and samples were de-identified. The neutrophil killing assay was performed as detailed previously (43). Purified neutrophils were incubated with mid-log-phase Strep A (OD_600_ = 0.4) at an MOI of 1:10 (Strep A: neutrophils) at 37°C + 5% CO_2_ for 1 h. After incubation, each replicate of experimental and negative-control groups was serially diluted in sterile water to lyse cells. All replicates and dilutions were plated on blood agar with results defined as %Strep A growth using the following formula: (CFU/mL in experimental group)/(CFU/mL in negative-control group) × 100%.

### Mice.

Specific pathogen-free 4- to 6-week-old BALB/c female mice were purchased from the Animal Resource Centre (Perth, Australia). All protocols were approved by Griffith University’s Animal Ethics Committee and were in accordance with National Health and Medical Research Council (NHMRC) guidelines.

### Antigen preparation and immunization regimen.

The peptides J8 (QAEDKVKQSREAKKQVEKALKQLEDKVQ) and K4S2 (KKKKNSDNIKENQFEDFDEDWENF) were synthesized by China Peptides Co. Ltd. (Shanghai, China). Each peptide was conjugated to DT via a C-terminal cysteine residue using 6′-maleimido-caproyl *N*-hydroxy succinimide, as described elsewhere (44).

The vaccine formulations were prepared fresh before each immunization by adsorbing DT-conjugated antigen with Alhydrogel (Alum; Brenntag Biosector, Denmark) as previously described (19). BALB/c mice were immunized with J8CombiVax (J8-DT+K4S2-DT/Alum) on days 0, 21, and 28 as previously described (11). Each mouse received 60 μg total vaccine formulation (30 μg J8 and 30 μg K4S2) per immunization. Control mice received adjuvant in PBS. Two weeks after the final immunization, mice were infected with Strep A via the superficial skin infection method.

### Superficial skin infection.

Mice were infected via the skin route as previously described (11). Briefly, mice were anesthetized via an intraperitoneal injection (100 μL/10 g mouse) of ketamine (100 mg/mL stock)/xylazil-20 (20 mg/mL stock)/water at a ratio of 1:1:10. Once fully anesthetized, the fur on the nape of the neck was removed to gently create a superficial skin wound and the bacterial inoculum (2 × 10^6^ CFU in 20 μL) was directly applied to the wound. Once the inoculum had absorbed, a temporary cover was applied, and mice were individually housed. Following infection, all mice were monitored daily.

### Tissue collection and processing.

On days 3 and 6 postinfection, a designated number of mice were culled via CO_2_ inhalation and blood, skin, and spleens were aseptically excised (11). Skin and spleen samples were homogenized using the Bullet Blender Homogenizer (Next Advance, NY, USA). Ten-fold serial dilutions of all samples were plated in duplicate on Columbia blood agar (Oxoid, Australia) with 5% defibrinated horse blood (Equicell, Australia) and incubated for 16 h at 37°C.

### Skin histology.

Skin samples were frozen in optimal cutting temperature (OCT) compound (Tissue-Tek; Sakura Finetek, CA, USA) for IHC and processed by the QIMR Berghofer histology facility. Scanned sections were analyzed at high magnification (20×) using ImageScope software (Leica Biosystems, Germany). Positively stained cells were quantified by counting five high-powered fields using ImageJ (NIH, USA).

### Statistics.

Statistical analyses were performed using one-way and two-way analyses of variance (ANOVAs) with multiple comparison post-tests in GraphPad Prism software version 6.0 (GraphPad, CA, USA). Where two groups were compared, Student’s *t* tests were used for analysis. A *P* value of <0.05 was considered statistically significant.

### Data availability.

Illumina Short reads were deposited to the NCBI SRA under The BioProject ID PRJNA926677 (SRR23196480 - SRR23196483).
