# Supplementary figures and images for: Streptolysin O Deficiency in Streptococcus pyogenes M1T1 covR/S Mutant Strain Attenuates Virulence in In Vitro and In Vivo Infection Models
Source: mBio. 2023 Feb 6;14(1):e03488-22. doi: 10.1128/mbio.03488-22 (PMC9972915; doi:10.1128/mbio.03488-22)

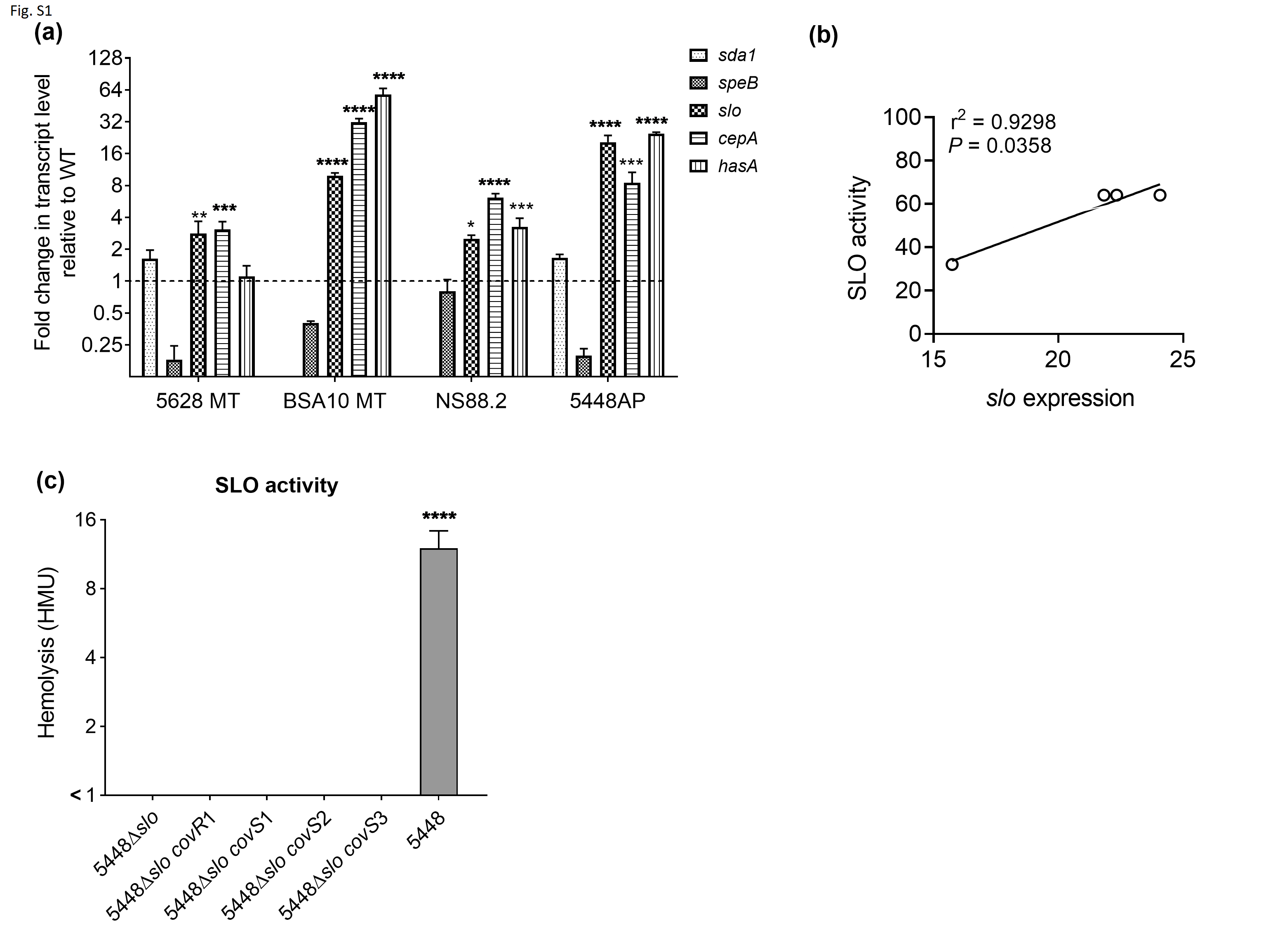

Supplement: FIG S1 [file mbio.03488-22-s0005.tif]

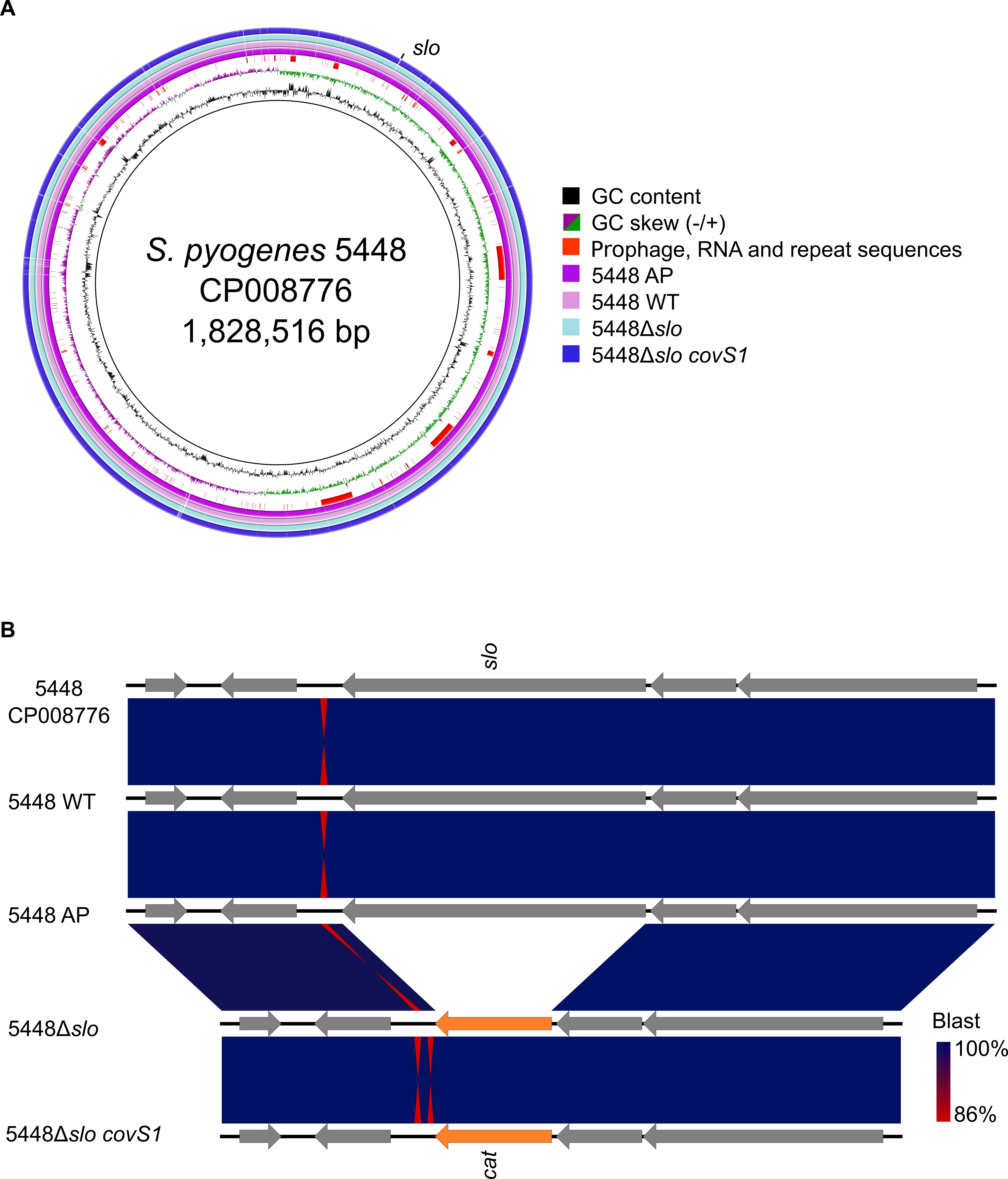

Supplement: FIG S2 [file mbio.03488-22-s0006.jpg]

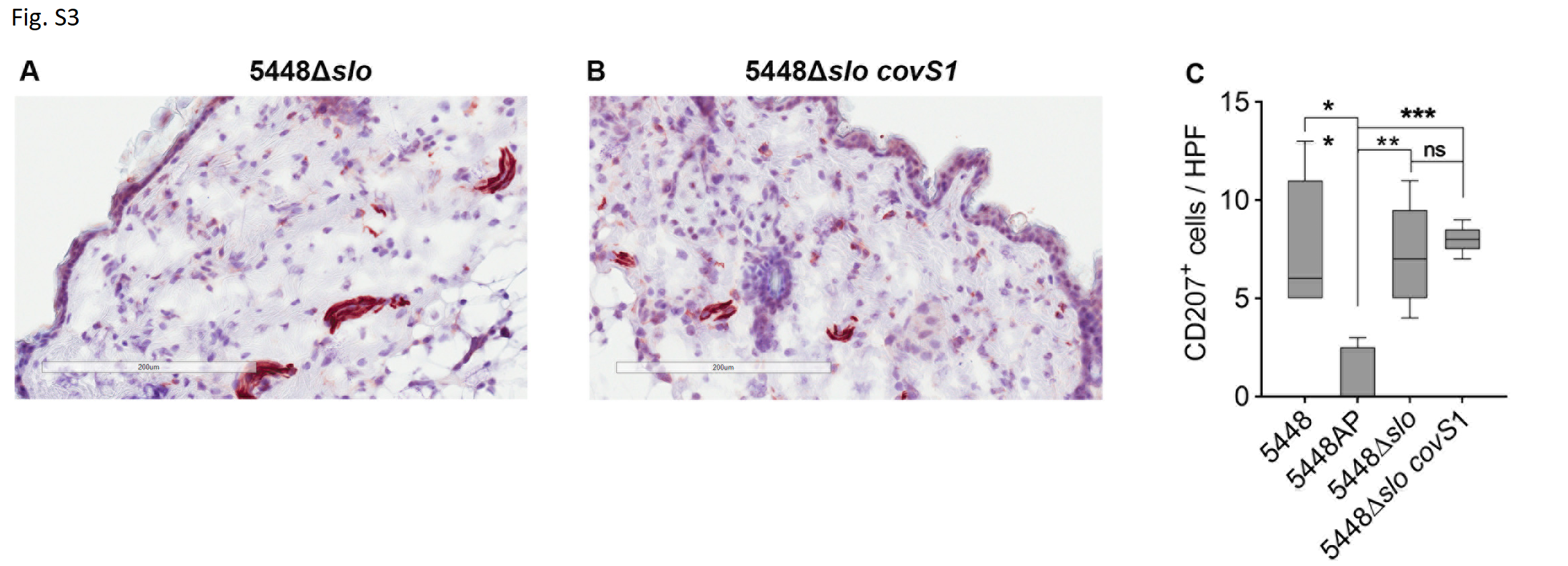

Supplement: FIG S3 [file mbio.03488-22-s0007.tif]

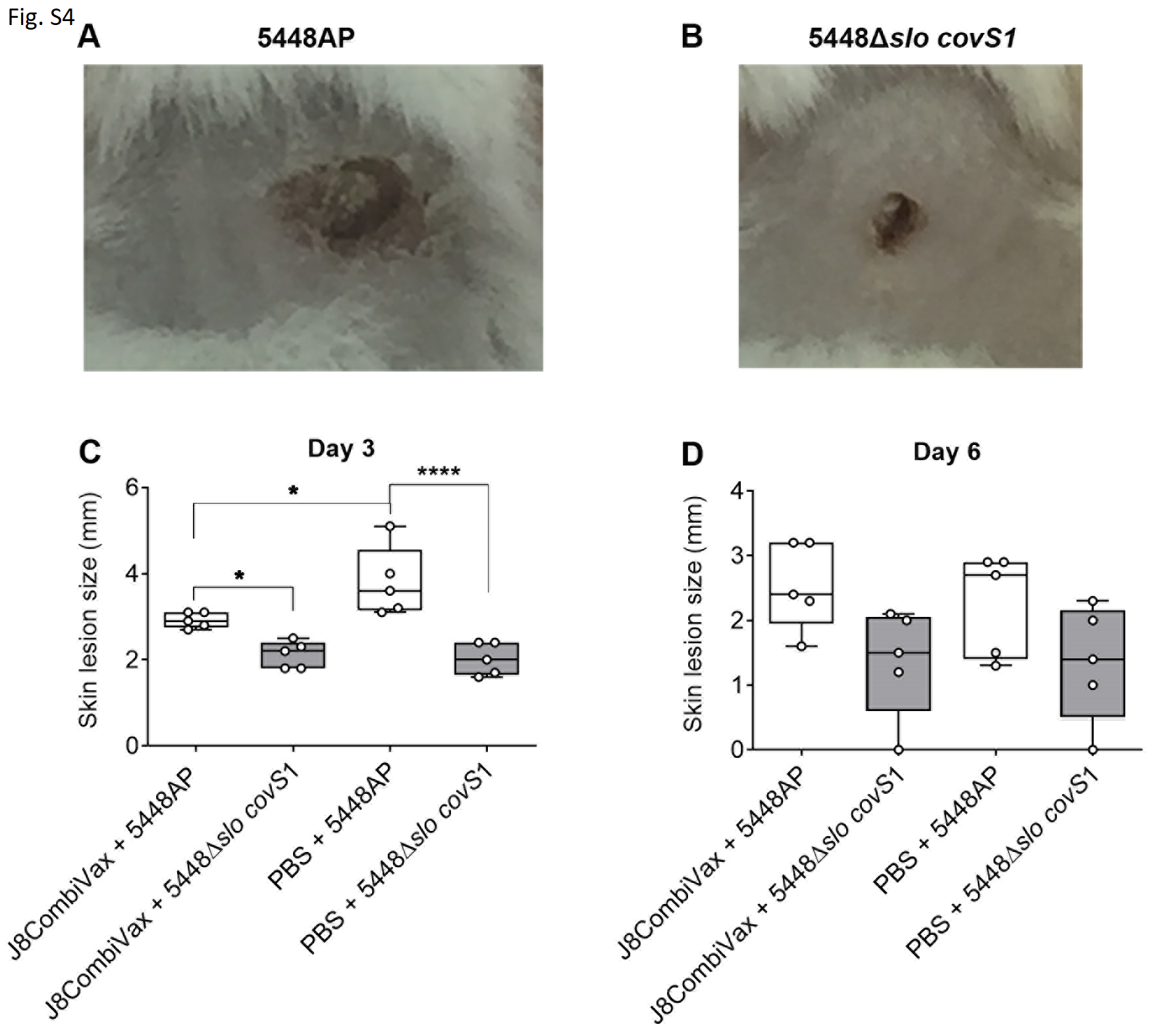

Supplement: FIG S4 [file mbio.03488-22-s0008.tif]
