# Supplementary material for: Streptolysin O Deficiency in Streptococcus pyogenes M1T1 covR/S Mutant Strain Attenuates Virulence in In Vitro and In Vivo Infection Models
Source: mBio. 2023 Feb 6;14(1):e03488-22. doi: 10.1128/mbio.03488-22 (PMC9972915; doi:10.1128/mbio.03488-22)
Supplement: TABLE S1 [file mbio.03488-22-s0001.pdf]

Table S1. 5448Δslo covR/S mutants[a].

**Name**	**Gene mutated**	**Mutation[b]**	**Length of operon[c]**
5448Δ*slo* *covR*1	*CovR*	G substituted for T at nt 341	*CovR* = 228 AA<br>*CovS* = 500 AA
5448Δ*slo* *covS*1	*CovS*	TT insertion at nt 473	*CovR* = 228 AA<br>*CovS* = 159 AA
5448Δ*slo* *covS*2	*CovS*	G substituted for T at nt 199	*CovR* = 228 AA<br>*CovS* = 66 AA
5448Δ*slo* *covS*3	*CovS*	C substituted for T at nt 9	*CovR* = 228 AA<br>*CovS* = 3 AA

[a] The 5448 *covR/S* sequencing details were as previously described (6).
[b] Mutation locations are based upon the nucleotide (nt) position in the *covR* or *covS* gene relative to the gene-specific ATG start codon.
[c] Length of operon is based upon the number of amino acids (AA) from the ATG start codon.
