# Supplementary material for: Streptolysin O Deficiency in Streptococcus pyogenes M1T1 covR/S Mutant Strain Attenuates Virulence in In Vitro and In Vivo Infection Models
Source: mBio. 2023 Feb 6;14(1):e03488-22. doi: 10.1128/mbio.03488-22 (PMC9972915; doi:10.1128/mbio.03488-22)
Supplement: TABLE S2 [file mbio.03488-22-s0002.pdf]

Table S2. SNP mutations in 5448 and 5448 mutants.

Position_in_5448	CDS/rRNA/tRNA/Intergenic	gene_name	CDS_name	product	Synonymous/Non-synonymous	Reference_base	SNP_base	5448Δ*slo*	5448Δ*slo covS* 1	5448AP	5448
86448	CDS	dexB	SP5448_00585	transglycosylase	Synonomous	G	T	T	T	T	T
147844	Intergenic		-	-	Intergenic	T	C	C	C	.	.
296507	CDS	dnaQ	SP5448_01640	DNA polymerase III subunit epsilon	Non-Synonomous	T	A	A	A	A	A
1578661	CDS	hsdM	SP5448_08275	restriction endonuclease subunit M	Non-Synonomous PREMATURE STOP	G	T	T	T	.	.
1633730	CDS	dexB	SP5448_08550	glucan 1,6-alpha-glucosidase	Non-Synonomous	G	T	.	.	.	T
1543856	CDS	covS	SP5448_08090	histidine kinase	Truncated Frameshift	G	GAA	.	GAA	.	.
1543451	CDS	covS	SP5448_08090	histidine kinase	Truncated Frameshift	A	AT	.	.	AT	.
