# Supplementary material for: Streptolysin O Deficiency in Streptococcus pyogenes M1T1 covR/S Mutant Strain Attenuates Virulence in In Vitro and In Vivo Infection Models
Source: mBio. 2023 Feb 6;14(1):e03488-22. doi: 10.1128/mbio.03488-22 (PMC9972915; doi:10.1128/mbio.03488-22)
Supplement: TABLE S3 [file mbio.03488-22-s0003.pdf]

Table S3. RT-PCR primers.

Primer name	bp	Sequence (5' – 3')
GYR F	20	GAAGTGATCCCTGGACCTGA
GYR R	20	CCCGACCTGTTTGAGTTGTT
SPEB F	20	GTGGAGTCTCTGACGGCTTC
SPEB R	20	TGCCTACAACAGCACTTTGG
SLO F	22	AAACAAACCAGACGCGGTAGTC
SLO R	21	GACCTCAACCGTTGCTTTGTC
CEPA F	20	ACACGGTATGCATGTGACAG
CEPA R	24	GATAAAGAGTGATTCAGGTGATCC
HASA F	20	ATGCTGCAACAGGACATTTG
HASA R	20	TTAATGATTGAGCAGCACGC
SDA1 F	22	GAGAGCCACTGAATCCGACTAC
SDA1 R	22	TACTGCATCCCACCTTTACGAT
