# Supplementary material for: Streptolysin O Deficiency in Streptococcus pyogenes M1T1 covR/S Mutant Strain Attenuates Virulence in In Vitro and In Vivo Infection Models
Source: mBio. 2023 Feb 6;14(1):e03488-22. doi: 10.1128/mbio.03488-22 (PMC9972915; doi:10.1128/mbio.03488-22)
Supplement: TABLE S4 [file mbio.03488-22-s0004.pdf]

Table S4. Sequencing primers.

Primer name	bp	Sequence (5' – 3')
CovRS 1F	19	GCTATTCCGGTACAGGTCT
CovRS 2F	19	GTCAATGGTCGTGAAGGGT
CovRS 3F	22	GATGTCTATATTCGTTATCTCC
CovRS 4F	22	GATGATTTTTACCACAGATAAC
CovRS 5F	20	GCATATTGGTCTCTTACAAC
CovRS 6F	21	GCAAATTGTAGATGGGTATCA
CovRS 7R	20	GCGGAAAATAGCACGAATAC
CovRS 8R	20	AGGCAATCAGTGTAAAGGCA
CovRS 9R	21	CTTGTGCCAAATAACTCAACA
CovRS 10R	21	ATCAAAAGCCTGCTCAAATGA
CovRS 11R	21	CTTTCATGTCATCCATCATTG
CovRS 12R	19	TTGCTCTCGTGTGCCA TCT
